# *C. elegans* CYLC-2 localizes to sperm

**DOI:** 10.17912/micropub.biology.000314

**Published:** 2020-09-29

**Authors:** Amber R Krauchunas, Michael Werner, Nicholas Britt, Dawn S Chen, Amy S Maddox, Andrew Singson

**Affiliations:** 1 Department of Genetics and Waksman Institute, Rutgers University, Piscataway, NJ 08854; 2 Department of Biology, University of North Carolina at Chapel Hill, Chapel Hill, NC 27599; 3 Department of Molecular Biology and Genetics, Cornell University, Ithaca, New York 14853

**Figure 1. CYLC-2::mNeonGreen localizes to sperm and has no effect on sperm function:**
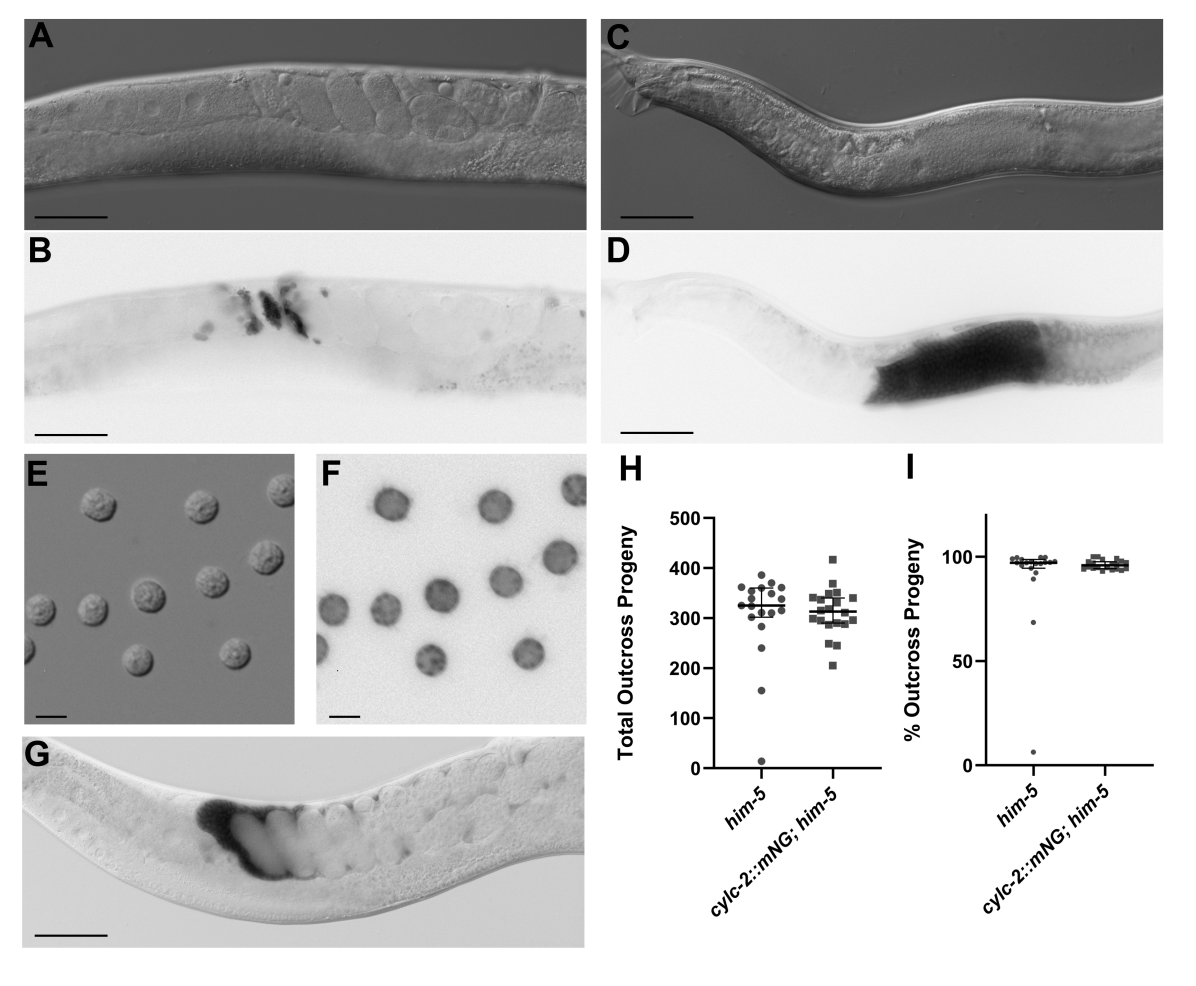
DIC and fluorescence images of young adult hermaphrodites (A-B), males (C-D), and dissected spermatids (E-F). Fluorescence signal clearly labels sperm in and near the spermatheca in the hermaphrodite and spermatids in the male. Scale bar, 50 µm (A-D). Scale bar, 5 µm (E-F). When AD294 males mate with N2 hermaphrodites, the CYLC-2::mNG labeled male sperm are visible in the hermaphrodite reproductive tract (G). Scale bar, 50 µm. Total outcross progeny (H) and percent outcross progeny (I) are comparable between control *him-5* males and *cylc-2::mNG; him-5* males indicating that tagging CYLC-2 with mNG has no effect on sperm formation or function. Median and 95% confidence intervals are shown in the graphs.

## Description

The gene products of *C. elegans* ORFs Y59E9AL.6 and C41G7.6 were assigned the names CYLC-1 and CYLC-2, respectively, due to their homology to human cylicins (Lacroix *et al.*. 2016). In mammals, expression of cylicin I and cylicin II is testis-specific and immunohistochemistry shows the proteins localize to the cytoskeletal calyx of the sperm head (Hess *et al.* 1993; Hess *et al.* 1995; Rousseaux-Prèvost *et al.* 2003). To determine the localization of *C. elegans* CYLC-1 and CYLC-2 we used CRISPR/Cas9 to endogenously tag each protein with mNeonGreen (mNG). We find that CYLC-2::mNG localizes to sperm in both hermaphrodites and males (Figure 1A-F). Examination of spermatids dissected from males shows CYLC-2::mNG concentrated in puncta (Figure 1F). Based on their size and position in the spermatids, we predict that these puncta correspond to the membranous organelles (MOs). However, further studies will be necessary to confirm whether CYLC-2 is concentrated in the MOs of spermatids and to determine if any changes in subcellular localization take place upon sperm activation.

We present CYLC-2::mNG as another genetically encoded sperm marker that can be used to track sperm presence, transfer, and movement (Figure 1G). For fertility studies it is often important to know if hermaphrodite self sperm are present in the spermathecae or if males have successfully mated and transferred sperm to a hermaphrodite. The creation of bright, sperm-specific fluorescent markers in *C. elegans* has been notoriously challenging (Wong *et al.*. 2020). Traditionally, the transfer and movement of sperm has been studied by indiscriminately marking male cells with vital dyes such as Mitotracker Red CMXRos or SYTO 17 (Hu *et al.* 2019; Singson *et al.*. 1999; Hill and L’Hernault 2001). Alternatively, fixation and staining with DAPI has been a blunt tool to confirm the presence or absence of sperm (Singaravelu *et al.* 2015; Krauchunas *et al.*. 2018). Most recently, the Stanfield lab has shown that combining two unlinked sperm histone::mCherry transgenes into the same strain significantly improved the fluorescent signal without impacting sperm function (Wong *et al.*. 2020).

CYLC-2::mNG is bright enough that it can be seen with just a fluorescence stereo microscope, allowing for the transfer of sperm to be monitored over time in free living hermaphrodites on a culture plate. It also makes crossing this sperm marker into mutant backgrounds relatively straightforward. Finally, it adds a bright, non-nuclear sperm marker to our community toolkit. By marking one population of sperm with a nuclear marker such as histone::mCherry and another with CYLC-2::mNG we can differentiate between hermaphrodite self sperm and male sperm, or sperm from two different males, within the hermaphrodite reproductive tract. There is no statistically significant difference in the total number of progeny sired by males with tagged CYLC-2 as compared to control males (p-value = 0.4, Figure 1H). In addition, there is no difference in the percentage of progeny produced that are outcross progeny (p-value = 0.58, Figure 1I). From these data, we infer that tagging CYLC-2 with mNG has no discernable effect on the ability of the sperm to migrate, compete with hermaphrodite sperm, or fertilize the egg.

We conclude that CYLC-2::mNG makes an excellent sperm marker to observe sperm transfer and migration in *C. elegans*. In addition, it is intriguing that the presence of these cylicin-like cytoskeletal proteins in sperm is conserved since *C. elegans* sperm have neither actin nor tubulin (L’Hernault and Roberts 1995). In the future, mutant studies will help us determine the role of cylicins in spermatogenesis, sperm structure, and sperm function.

## Methods

**Strains and Husbandry**

*C. elegans* strains were cultured at 20^o^C using standard methods (Brenner, 1974).

Strains used:

MDX44 *C41G7.6/cylc-2(mon2[C41G7.6::mNG^3xFLAG) I*

AD294 *C41G7.6/cylc-2(mon2[C41G7.6::mNG^3xFLAG) I; him-5(e1490) V*

N2 Bristol wild-type

CB61 *dpy-5(e61)*

mNG = mNeonGreen

**CRISPR/Cas9 genome editing to create *cylc-2::mNG***

CRISPR lines were generated using SEC (Self-Excising Cassette)-CRISPR system (Dickinson *et al.* 2015). In short, a guide sequence for C41G7.6 (gatgagaagaaggagtgagc) was introduced into pDD162 using the NEB Q5 site directed mutagenesis kit. 500-700 base pair repair constructs were generated by PCR and cloned into pDD268. The PAM site used for C41G7.6 was part of the guide sequence and was left unchanged in the repair sequence. After isolation of CRISPR insertion lines, verified lines were subjected to heat shock treatment to remove the SEC cassette.

**Imaging**

AD294 *c41g7.6(mon2[c41g7.6::mNG^3xFlag); him-5(e1490)* hermaphrodites and males were separated from one another at the L4 stage and imaged the following day. Whole live worms were mounted in M9 with levamisole on 2% agarose pads. To observe transfer of marked sperm to unmarked hermaphrodites, several AD294 males were placed with several N2 L4 hermaphrodites. The hermaphrodites were mounted and imaged approximately 24 hours later. To observe isolated spermatids, virgin males were dissected in Sperm Medium (10 mM dextrose, 1 mM MgSO_4_, 5 mM CaCl_2_, 50 mM NaCl, 25 mM KCl, and 5.5 mM HEPES pH 7.8). Differential interference contrast microscopy (DIC) and fluorescent images of live worms or dissected spermatids were obtained using a Zeiss Universal microscope and captured with a ProgRes camera (Jenoptik) using ProgResCapturePro software.

**Male fertility assay**

Crosses were set up between *dpy-5(e61)* L4 hermaphrodites and either *him-5(e1490)* orAD294 *c41g7.6/cylc-2(mon2[c41g7.6::mNG^3xFlag); him-5(e1490)* males in a 1:4 ratio. 24 hours later, the males were removed and the hermaphrodites were transferred to new plates. The hermaphrodites continued to be transferred to new plates every 24 hours for a total of 4 days. On the last day the hermaphrodites were removed but not transferred to new plates. Three days after the hermaphrodite was removed from the plate, the number of Dpy and non-Dpy progeny was counted. Outcross progeny is the total number of non-Dpy progeny produced by a single hermaphrodite during the four days. Percent outcross progeny is the total number of non-Dpy progeny divided by the total number of progeny (Dpy + non-Dpy) produced by a single hermaphrodite during the four days. Mann-Whitney tests were performed to determine if there were statistically significant differences in the total number of outcross progeny or the percent outcross progeny.
